# Supplementary material for: Spike S2 Subunit: Possible Target for Detecting Novel SARS-CoV-2 Variants with Multiple Mutations
Source: Trop Med Infect Dis. 2024 Feb 15;9(2):50. doi: 10.3390/tropicalmed9020050 (PMC10893286; doi:10.3390/tropicalmed9020050)
Supplement: Supplementary file 1 [file tropicalmed-09-00050-s001.zip › Supplementary table.pdf]

Table S1. Sequences of SARS-CoV-2 genome submitted from January to October 2022

Country	Number of submitted virus	Start date	End date
**USA**	61596	Jan 5, 2022	Oct 2, 2022
**Canada**	22234	Jan 5, 2022	Oct 2, 2022
**Australia**	5961	Jan 5, 2022	Oct 2, 2022
**Germany**	60955	Jan 5, 2022	Oct 2, 2022
**France**	7930	Jan 5, 2022	Oct 2, 2022
**England**	19110	Jan 5, 2022	Oct 2, 2022
**Italy**	9204	Jan 5, 2022	Oct 2, 2022
**Russia**	1191	Jan 5, 2022	Oct 2, 2022
**China**	143	Jan 5, 2022	Oct 2, 2022
**Japan**	6815	Jan 5, 2022	Oct 2, 2022
**South Korea**	5014	Jan 5, 2022	Oct 2, 2022
**India**	12353	Jan 5, 2022	Oct 2, 2022
**Iceland**	129	Jan 5, 2022	Oct 2, 2022
**Brazil**	1476	Jan 5, 2022	Oct 2, 2022
**Spain**	6206	Jan 5, 2022	Oct 2, 2022
**Belgium**	7731	Jan 5, 2022	Oct 2, 2022
**Turkey**	936	Jan 5, 2022	Oct 2, 2022
**Peru**	272	Jan 5, 2022	Oct 2, 2022
**Chile**	408	Jan 5, 2022	Oct 2, 2022
**Thailand**	1157	Jan 5, 2022	Oct 2, 2022
**Total**	**230,821**		

Table S2: Mutations in the binding sites of primers and probe.

Primer	Lineage	Country	Mutation in primer	Mutation in 3' end	Number sequences	Frequency (Total 230,821)
S2F	BA.1.1.7	India	18	5	1	0.0004%
	Unassigned	India	5	5	1	0.0004%
	B.1.1	Turkey	4	4	1	0.0004%
	B.1.1	Turkey	3	3	1	0.0004%
	BA.1	Turkey	3	2	1	0.0004%
	B.1.1	Turkey	2	2	1	0.0004%
	BA.5.3.2	Germany	4	1	1	0.0004%
			1	1	65	0.0282%
			5	0	6	0.0026%
			3	0	1	0.0004%
			2	0	1	0.0004%
			1	0	250	0.1083%
				**Total**	**330**	**0.1430%**
S2R	Unassigned	USA	4	4	1	0.0004%
	B.1.1.529	USA	4	3	1	0.0004%
	BA.2	Turkey	3	3	1	0.0004%
	BA.1	Turkey	3	1	1	0.0004%
			1	1	171	0.0741%
			5	0	7	0.0030%
			4	0	1	0.0004%
			3	0	3	0.0013%
			2	0	2	0.0009%
			1	0	179	0.0775%
				**Total**	**367**	**0.1590%**
S2Pr	BA.1	Turkey	4	4	1	0.0004%
	BE.1	Turkey	3	3	1	0.0004%
	BA.2.12.1	USA	3	3	1	0.0004%
	BA.1	USA	2	2	1	0.0004%
			1	1	44	0.0191%
			2	0	5	0.0022%
			1	0	1085	0.4701%
				**Total**	**1138**	**0.4930%**
