# Supplementary figures and images for: Spike S2 Subunit: Possible Target for Detecting Novel SARS-CoV-2 Variants with Multiple Mutations
Source: Trop Med Infect Dis. 2024 Feb 15;9(2):50. doi: 10.3390/tropicalmed9020050 (PMC10893286; doi:10.3390/tropicalmed9020050)

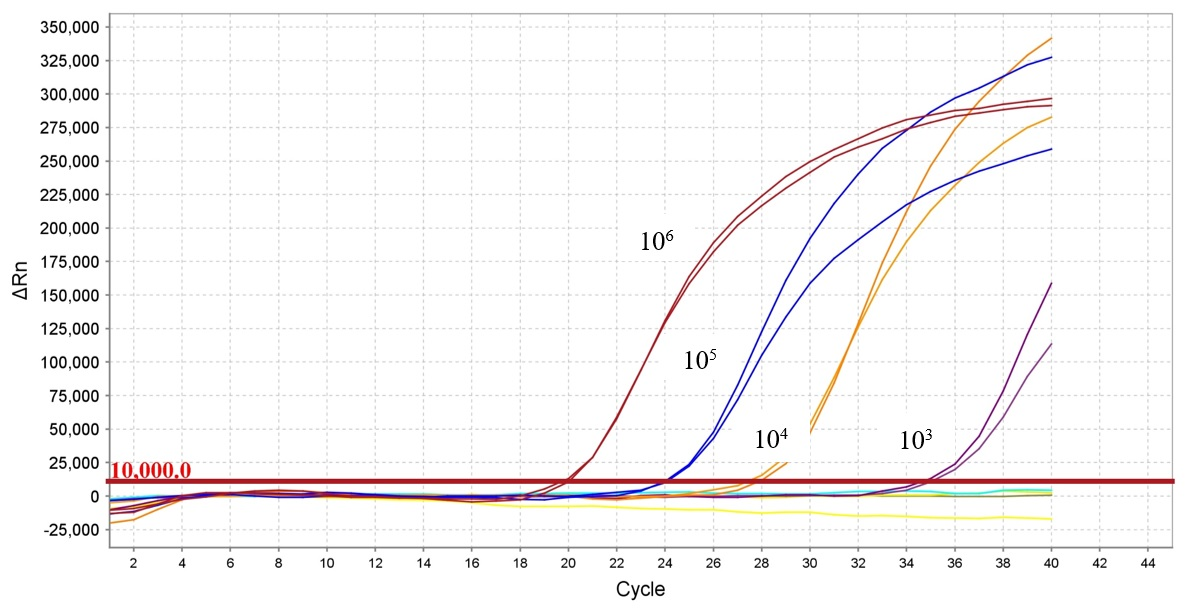

Supplement: Supplementary file 1 [file tropicalmed-09-00050-s001.zip › Figure S1.png]
